# Fractal-fractional modeling and stability analysis of pine wilt disease dynamics

**DOI:** 10.1371/journal.pone.0318534

**Published:** 2025-02-12

**Authors:** Khaled Aldwoah, Shahid Ahmed, Shah Jahan, Amel Touati, Nidal EIjaneid, Tariq AIjaaidi

**Affiliations:** 1 Department of Mathematics, Faculty of Science, Islamic University of Madinah, Medinah, Saudi Arabia; 2 Department of Mathematics, Central University of Haryana, Mahendergarh, India; 3 Department of Mathematics, Faculty of Science, Northern Border University, Arar, Saudi Arabia; 4 Department of Mathematics, Faculty of Science, University of Tabuk, Tabuk, Saudi Arabia; 5 Department of Artificial Intelligence, Faculty of Computer Science and Information Technology, Alrazi University, Sana’a, Yemen; University of Porto Faculty of Engineering: Universidade do Porto Faculdade de Engenharia, PORTUGAL

## Abstract

In this article, we have constructed a compartmental mathematical model employing fractal-fractional operators to investigate the dynamics of pine wilt disease. The model comprises six nonlinear ordinary differential equations, representing six compartments for individuals categorized as susceptible, exposed, and infected. Furthermore, we restructured the model by applying methodologies that are based on fractional calculus and fractal theory, one can gain significant insights into the intricacies of pine wilt disease transmission. The model’s essential properties, that is existence and uniqueness were analysed using the Banach and Leray-Schauder theorems. We study the stability of the fractional model by applying the Ulam-Hyers stability conditions. Additionally, computational techniques for the model in fractal-fractional cases are formulated using an iterative numerical approach like the fractional Adams-Bashforth methodology. Finally, we presented a comprehensive simulation conducted to validate the theoretical findings. The results are simulated to correspond to various fractional order values (*θ*_1_) and fractal dimensions (*θ*_2_) using MATLAB.

## 1 Introduction

Mathematical models are extensively employed in epidemiology to gain a deeper understanding of infectious illness dynamics [1]. Such models are not confined solely to human health but find wide-ranging applications in various aspects of biological sciences, encompassing ecology and forestry, among others. Forests play a pivotal role in human life, so it becomes imperative to implement effective strategies for safeguarding them against disease outbreaks. Forests not only contribute to the greenery of our environment but also create a pleasant atmosphere for humans to enjoy. Pine wilt disease(PWD) a devastating ailment affecting pine trees, stands out as a significant threat to both ecosystems and forests. PWD is an alarming disease that, in a matter of weeks or months, can quickly cause affected trees to die. The culprit behind this devastating ailment is the pine tree nematode, scientifically known as Bursaphelenchus xylophilus [2]. In contrast to needle diseases, the progression of tree mortality typically begins in the upper portions of the tree and gradually extends downward. The transmission of these pinewood nematodes takes place during the spring season, when infected pine sawyer beetles act as carriers, transferring the pathogen from diseased pine trees to healthy ones. Furthermore, it’s worth noting that mathematical models have shown an important role in the field of epidemiology [3]. These models are commonly employed to gain insights into the dynamics of infectious diseases [4, 5]. These applications extend beyond human health and find relevance in various physical contexts, including ecosystems like forests. Forests are a vital component of our daily lives, and it is imperative that we take concerted efforts to safeguard them against the infectious threat of deforestation. Among the various problems that forests and ecosystems encounter, PWD is a major and threatening issue. The PWD is a devastating problem that can quickly kill pine trees. Telltale signs, such as needle discoloration the transition from yellow to green and then to a reddish-brown hue signifies the onset of the condition. The primary culprit behind this ailment is a tiny worm known as the pinewood nematode, and its presence leads to the gradual decline of the affected tree [6, 7]. We can classify the primary actors in the PWD ecosystem into three key components: the pine wood nematode, the gymnosperm host, and the insect vector. Recently, several models have been proposed to thoroughly examine this disease’s behaviour using a set of nonlinear differential equations for analysis [8]. Prior research has demonstrated that mathematical models incorporating fractional calculus (FC) techniques often demonstrate enhanced accuracy and stability compared to those relying solely on integer-order calculus. This advantage arises from the increased degrees of freedom inherent in FC [9]. However, it is important to recognize that a considerable portion of biological models continue to rely on classical methodologies, giving rise to systems of nonlinear differential equations. This suggests that there is untapped potential for improving mathematical models through the use of advanced FC techniques [10]. Recent studies involving a range of biological models have highlighted the significant usefulness and improved precision of these methods in comparison to traditional ones. Ahmad et al. [11] discussed the sensitivity analysis of PWD via fractal-fractal derivatives of the Mittag-Leffler kernel. Atangana et al. [12] discussed the concept of beta-derivative to fractional model for Rubella disease. They also provided evidence of its stability and independence. Qureshi et al. [13] examined a study of ordinary and FO models related to the dengue epidemic. Muhammad et al. [14] studied the mathematical modeling of the infectious Ebola disease, employing operators such as Caputo-Fabrizio, Caputo, and Atanagana-Baleanu to gain insights into the disease’s dynamics. Recently, Ahmed et al. [15] discussed the fractional approach to modeling and analyzing a system of lakes affected by pollution. Zhou et al. [16] used the fractal-fractional(FF) operator for COVID-19 modeling and dynamics. Shaikh et al. [17] used fractional derivatives with the Mittag-Leffler kernel for the dynamics of HIV/AIDS in 2022. The inclusion of fractional derivatives introduces memory and heritability effects into the model, which makes it possible to show problems more accurately and capture the complex ways that epidemics change over time. In 2017, Atangana [18] introduced novel FF derivatives, which exhibit crossover behavior. These operators combine established fractional and fractal calculi, offering a versatile framework for modeling complex phenomena. Additionally, computational transmission models employing fractional derivatives have been developed to analyze the dynamics and control strategies of diseases like HIV/AIDS [19], zoonotic [20], and monkeypox [21], providing valuable insights into disease transmission dynamics and intervention planning.

Stability is a critical aspect of qualitative theory in relation to differential equations. It is well- recognised that, on certain occasions, obtaining an exact solution can prove to be exceedingly challenging. Consequently, researchers have developed several numerical methods to tackle this challenge. In this context, we focus on assessing the stability of the PWD model 1. In existing literature, various forms of stability are described, which encompass Lyapunov [22], exponential and asymptotic stability [17], among others. Nonetheless, Ulam proposed one of the most important types of stability in 1940, known as Ulam stability [22, 23]. He asked a question pertaining to the stability of functional equations. The following year, Hyers provided a partial response to Ulam’s inquiry within the context of Banach spaces [22]. Researchers have further expanded and generalized Hyers results in various directions, encompassing both difference and functional equations. Jung et al. [24] use UHS of linear difference equations in Banach spaces. Baisas et al. [25], on the Ulam stability of linear equations. Wang et al. [26], on the existence and UHS of fractional coupled equations. Numerical and optimization aspects emphasize the critical importance of Ullam-Hyers stability(UHS) as It acts as a pivotal link connecting exact and numerical solutions. Given the complexities and prevalence of misinformation surrounding PWD, building an appropriate mathematical model using classical order differentiation can be challenging. In such circumstances, nonlocal operators provide a beneficial approach because they can effectively account for non-local phenomena and incorporate memory effects. These memory effects may vary based on the existence of power-law behaviour, fading memory, or crossover effects. Nonetheless, in some cases, the combination of power-law behavior, fading memory, and crossover effects may not adequately describe the detailed dynamics found in the viral illness. In such cases, recently introduced operators that encompass both fractal and fractional orders can serve as more appropriate mathematical tools for addressing these complex behaviors. FF operators have given new directions in modeling approaches for analyzing complex problems. Atangana et al. [27] discussed some misinterpretations and lack of understanding of differential operators. Zhou et al. [16], modeling the dynamics of COVID-19 using a FF operator. Atangana et al. [18] proposed a novel set of operators termed FF differential and integral operators. These operators establish a link between FC and fractal calculus. Notably, these recently introduced operators possess two distinct components: the first pertains to a FO, while the second corresponds to the FD. Subsequent research has demonstrated that these FF order operators prove to be highly effective tools for analyzing mathematical models involving real-world data [17, 28]. For instance, Aguilar et al. [29] applied these operators to investigate the transmission dynamics of malaria. Atangana et al. [30] discussed the modeling and analysis of nonlinear dynamics in the transmission of diarrhea. Li et al. [31] used the FF operator for investigating bank data. Zarin et al. [15] studied the FO dynamics of an epidemic model that combines Chagas and HIV, exploring various fractional operators. The intrinsic memory effect is a common characteristic among fractional derivatives and integrals. Haidong et al. [32] discussed system of Typhoid disease including protection from infection using fractal fractional operator, also Rahman et al. [33, 34] applied the generalized fractal fractional order problems under Mittag Leffler kernel and mathematical insights of social media addiction using fractal fractional perspectives. Li et al. [35] investigation of financial bubble mathematical model under fractal fractional. Torab et al. [36] the exitence results of solutions for nonlinear fractional boundary problem and also Torab et al [37] discussed the solability of a non linear langevin equations involving two fractional orders in different interval. Zhu et al. [38] disccused Ensemble classifier design based on swarm algorithm and Ahmed et al. [39] applied Caputo fractional order ebola virus model for control measures. This characteristic serves as the primary motivation for the PWD model’s dynamics using an effective FF operator. Fractal fractional order derivatives provide a powerful framework for understanding disease dynamics and designing effective eradication strategies by capturing the complex, scale-invariant processes inherent in biological systems and incorporating memory effects into mathematical models [21, 40]. Their integration into epidemiological modeling can lead to more accurate predictions and improved control measures for combating infectious diseases [19, 28, 41]. Our objective is to deepen the understanding of the unique dynamics within the PWD model by leveraging the core principles of the FF operator to generate significant numerical outcomes. We build on earlier research [8, 42] by examining the model’s properties, such as uniqueness, existence, and stability, through mathematical analysis. We also develop computer programs specifically designed for fractal-fractional cases, allowing us to test theories using detailed simulations that work with different fractional orders and fractal dimensions. This approach is a major step forward in modeling infectious diseases, as it enables the exploration of complex dynamics and the evaluation of intervention strategies in a more realistic way. The paper primarily demonstrates how fractional calculus and fractal theory can be applied to model infectious diseases. It also shows how advanced computing techniques can help us learn more about how diseases spread and how to make public health policies and programs better.

The rest of the article is organized as follows: Section 2 describes the PWD model and its fractional counterpart, utilizing the FF Caputo operator. Section 3 elaborates on the theoretical properties associated with the FF model, including the existence and uniqueness analysis. Section 4 establishes the necessary criteria to ensure the UHS of the model solution. Section 5 presents the numerical scheme for the PWD model using the FF operator. Section 6 includes the numerical findings and an explanation of the numerical approach, which is then implemented using MATLAB. Finally, the conclusion is presented.

## 2 PWD model description

In this section, we discussed the dynamic characteristics of the PWD model, as discussed in [8, 42].

The model incorporates several key parameters to describe various interactions and processes:

- The parameter *M*1 quantifies the contact rate between susceptible pine trees and infected vectors at a given time *t*.
- Parameter *M*2 represents the contact rate between susceptible pine trees and infected vectors during nematode transmission, specifically during oviposition.
- Ξ_*h*_ and Ξ_*v*_ signify the recruitment rates for pine trees and vector populations.
- Parameters *η*_1_ and *η*_2_ serve as saturation constants governing these recruitment processes.
- Natural death rates for the pine tree and vector populations is denoted by ϒ_*h*_ and ϒ_*v*_, respectively.
- Natural death rate of the populations is symbolized by Ω.
- *π*_1_ signifies the contact rate between infected trees and susceptible vector beetles.
- Λ_*h*_ and Λ_*v*_ represent the rates of transition from the exposed class to the infected class for pine trees and vector populations, respectively.

These parameters collectively define the dynamics of the model, describing the interactions and vital processes within the pine tree and vector populations in the context of PWD. The total population of pine trees is defined as $N_{h}\left(t\right)$, where $N_{h}\left(t\right)=S_{h}\left(t\right)+E_{h}\left(t\right)+I_{h}\left(t\right)$. In this model, the symbol $S_{h}\left(t\right)$ represent the class of susceptible pine trees at time *t*, $E_{h}\left(t\right)$, and $I_{h}\left(t\right)$ represent the class of exposed, and infected pine trees at time *t* respectively. Similarly, the symbol $S_{v}\left(t\right)$ is for susceptible, $E_{v}\left(t\right)$, and $I_{v}\left(t\right)$ are used to denote the exposed, and infected class of vectors at the same time *t*. The nonlinear system of ordinary differential equations is expressed as follows:

$$\begin{array}{c} \left\{ \begin{array}{c} \frac{dS_{h}\left(t\right)}{dt}=\Xi_{h}-\frac{M_{1}S_{h}\left(t\right)I_{v}\left(t\right)}{1+\eta_{1}I_{v}\left(t\right)}-\frac{M_{2}\Omega S_{h}\left(t\right)I_{v}\left(t\right)}{1+\eta_{1}I_{v}\left(t\right)}-{ϒ}_{h}S_{h}\left(t\right)\\ \frac{dE_{h}\left(t\right)}{dt}=\frac{M_{1}S_{h}\left(t\right)I_{v}\left(t\right)}{1+\eta_{1}I_{v}\left(t\right)}-{ϒ}_{h}E_{h}\left(t\right)-\Lambda_{h}E_{h}\left(t\right)\\ \frac{dI_{h}\left(t\right)}{dt}=\frac{M_{2}\Omega S_{h}\left(t\right)I_{v}\left(t\right)}{1+\eta_{1}I_{v}\left(t\right)}+\Lambda_{h}E_{h}\left(t\right)-{ϒ}_{h}I_{h}\left(t\right)\\ \frac{dS_{v}\left(t\right)}{dt}=\Xi_{v}-\frac{\pi_{1}S_{v}\left(t\right)I_{h}\left(t\right)}{1+\eta_{2}I_{h}\left(t\right)}-{ϒ}_{v}S_{v}\left(t\right)\\ \frac{dE_{v}\left(t\right)}{dt}=\frac{\pi_{1}S_{v}\left(t\right)I_{h}\left(t\right)}{1+\eta_{2}I_{h}\left(t\right)}-{ϒ}_{v}E_{v}\left(t\right)-\Lambda_{v}E_{v}\left(t\right)\\ \frac{dI_{v}\left(t\right)}{dt}={ϒ}_{v}E_{v}\left(t\right)-\Lambda_{v}I_{v}\left(t\right). \end{array}\right. \end{array}$$ (1)

**Fig 1:**
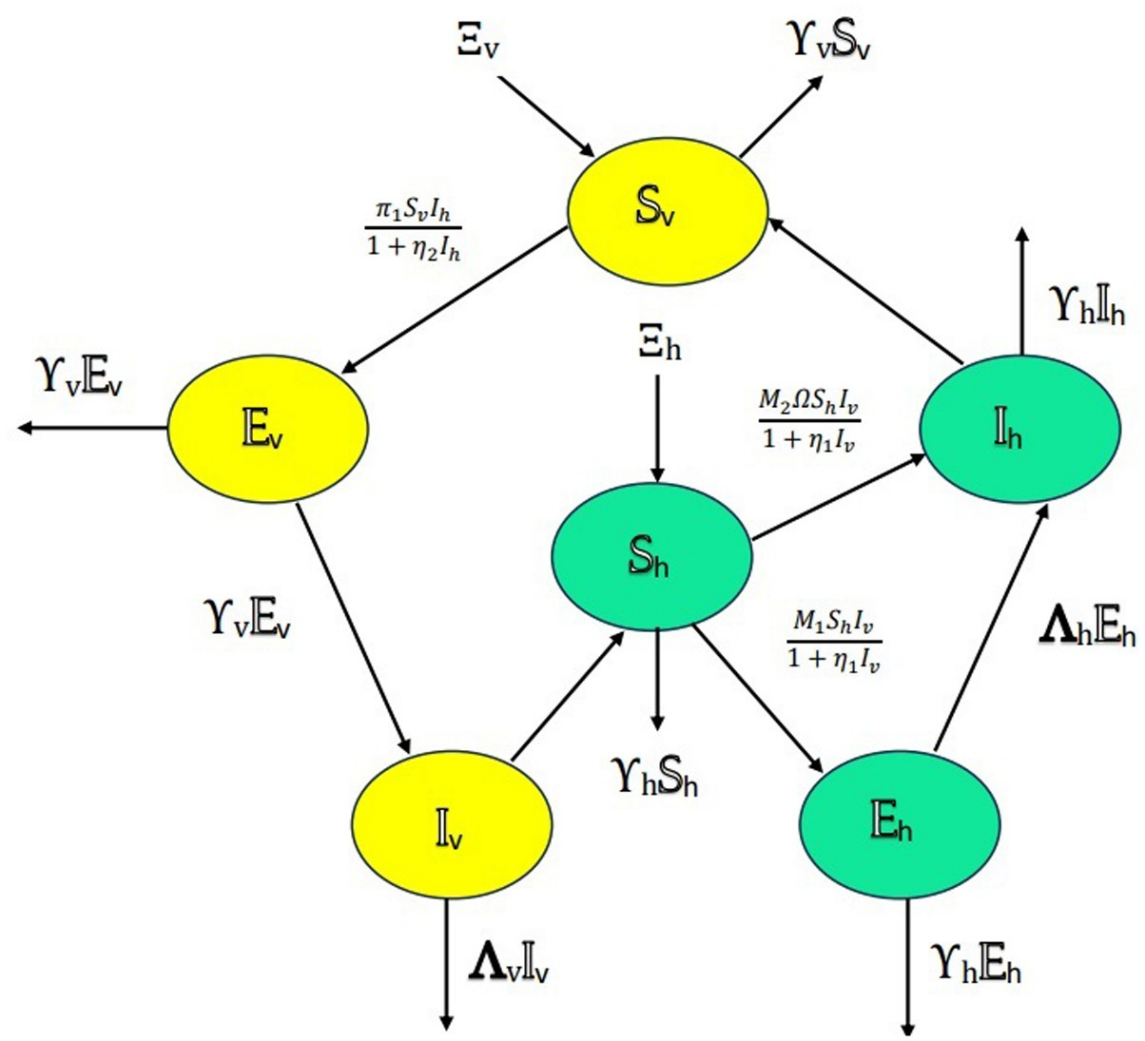
Schematic diagram for PWD.

Here we provide an explanation for each of the parameters in the model (1): These parameters collectively define the dynamics of the model (1) and play an important part in describing the interactions and processes within the system involving pine trees, vectors, and nematodes. The inclusion of fractal-fractional order derivatives in mathematical models of disease dynamics offers significant benefits, including better representation of complex structures, accounting for memory effects, flexibility in model adaptation, and enhanced predictive capability. By adjusting the fractional and fractal order parameters, we can tailor the model to reflect the specific characteristics of the disease and population under study. Fig 1 illustrates the schematic diagram for the PWD model, describing the various dynamics and interactions between pine trees, vectors, and nematodes. This model can be used to understand the spread of the disease and its impact on tree populations over time.

### 2.1 Preliminaries

**Definition 2.1** [16] *Let ϑ*(t) *be a function on the open interval* (a, b), *characterized by continuity and differentiability up to order θ*_2_. *The FF derivative of ϑ*(t) *with a θ*_1_ *order Riemann-Liouville (R-L) derivative is given by*:

DFFPθ1,θ2ϑ(t)=1Γ(n-θ1)ddtθ2∫0t(t-z˜)n-θ1-1ϑ(z˜)dz˜, (2)

*with n* − 1 < *θ*_1_, *θ*_2_ ⩽ *n, where* n∈N *and* $\frac{d\vartheta (\tilde{z})}{d{\tilde{z}}^{\theta_{2}}}={\text{lim}}_{t\to \tilde{z}}\frac{\vartheta \left(t\right)-\underline{\vartheta }\left(\tilde{z}\right)}{t^{\theta_{2}}-{\tilde{z}}^{\theta_{2}}}$. *The function ϑ*(t) *is defined on the open interval* (a, b), *meaning it is only considered for values of* t *within this range. This function is assumed to be continuous and differentiable up to a certain order, denoted by θ*_2_. *Continuity ensures that the function doesn’t have any breaks or jumps in this interval, while differentiability up to order* *θ*_2_ *implies that ϑ*(t) *has well-defined derivatives up to that order*.

**Definition 2.2** [16] *On the interval* (*a*, *b*), *assuming that the function ϑ*(*t*) *is continuous, the expression for the FF integral of ϑ*(*t*) *with order θ*_1_ *can be stated as follows*:

IFFPθ1ϑ(t)=θ2Γ(θ1)∫0t(t-z˜)θ1-1z˜θ2-1ϑ(z˜)dz˜. (3)

*θ*_1_ depicts how much influence past values of the function *ϑ*(t) have on its current rate of change. *θ*_2_ controls the fractal aspect, capturing the complexity and self-similar patterns in the system’s behavior.

**Note:** Let us define Banach space **V** = **W** × **W** × **W** × **W** × **W** × **W**, where **W** = *C*(**I**) under the norm: $\left\|Z\|=\right\|S_{h},E_{h},I_{h},S_{v},E_{v},I_{v}\|={\text{max}}_{t\in S}\left[\right|S_{h}\left(t\right)|+|E_{h}\left(t\right)|+|I_{h}\left(t\right)|+|S_{v}\left(t\right)|+|E_{v}\left(t\right)|+|I_{v}\left(t\right)\left|\right]$. The vector **Z** is composed of the functions $S_{h}\left(t\right)$, $E_{h}\left(t\right)$, $I_{h}\left(t\right)$, $S_{v}\left(t\right)$, $E_{v}\left(t\right)$, and $I_{v}\left(t\right)$, which together describe the state of the human and vector populations over time. The norm is defined as the maximum value, over the interval **S**, of the sum of the absolute values of these six variables, it measures the peak combined magnitude of these populations at any time t within the interval being studied.

## 3 Qualitative analysis

In this section, we delve into the existence and uniqueness of the model described by Eq (1). Before exploring into an analysis of a biological model, it is crucial to ascertain the actual existence of such a dynamic problem in the real world. Fixed point theory is utilized to evaluate the system described by Eq (1) and determine its uniqueness. Similarly, we plan to apply this theory to the model represented by Eq (1). Because of the integral’s differentiability, the problem (1) can be transformed as follows:

{DRLθ1Sh(t)=qtθ2-1g1(t,Sh,Eh,Ih,Sv,Ev,Iv)DRLθ1Eh(t)=qtθ2-1g2(t,Sh,Eh,Ih,Sv,Ev,Iv)DRLθ1Ih(t)=qtθ2-1g3(t,Sh,Eh,Ih,Sv,Ev,Iv)DRLθ1Sv(t)=qtθ2-1g4(t,Sh,Eh,Ih,Sv,Ev,Iv)DRLθ1Ev(t)=qtθ2-1g5(t,Sh,Eh,Ih,Sv,Ev,Iv)DRLθ1Iv(t)=qtθ2-1g6(t,Sh,Eh,Ih,Sv,Ev,Iv), (4)

where

$$\begin{array}{c} \left\{ \begin{array}{c} g_{1}\left(t,S_{h},E_{h},I_{h},S_{v},E_{v},I_{v}\right)=\Xi_{h}-\frac{M_{1}S_{h}\left(t\right)I_{v}\left(t\right)}{1+\eta_{1}I_{v}\left(t\right)}-\frac{M_{2}\Omega S_{h}\left(t\right)I_{v}\left(t\right)}{1+\eta_{1}I_{v}\left(t\right)}-{ϒ}_{h}S_{h}\left(t\right)\\ g_{2}\left(t,S_{h},E_{h},I_{h},S_{v},E_{v},I_{v}\right)=\frac{M_{1}S_{h}\left(t\right)I_{v}\left(t\right)}{1+\eta_{1}I_{v}\left(t\right)}-{ϒ}_{h}E_{h}\left(t\right)-\Lambda_{h}E_{h}\left(t\right)\\ g_{3}\left(t,S_{h},E_{h},I_{h},S_{v},E_{v},I_{v}\right)=\frac{M_{2}\Omega S_{h}\left(t\right)I_{v}\left(t\right)}{1+\eta_{1}I_{v}\left(t\right)}+\Lambda_{h}E_{h}\left(t\right)-{ϒ}_{h}I_{h}\left(t\right)\\ g_{4}\left(t,S_{h},E_{h},I_{h},S_{v},E_{v},I_{v}\right)=\Xi_{v}-\frac{\pi_{1}S_{v}\left(t\right)I_{h}\left(t\right)}{1+\eta_{2}I_{h}\left(t\right)}-{ϒ}_{v}S_{v}\left(t\right)\\ g_{5}\left(t,S_{h},E_{h},I_{h},S_{v},E_{v},I_{v}\right)=\frac{\pi_{1}S_{v}\left(t\right)I_{h}\left(t\right)}{1+\eta_{2}I_{h}\left(t\right)}-{ϒ}_{v}E_{v}\left(t\right)-\Lambda_{v}E_{v}\left(t\right)\\ g_{6}\left(t,S_{h},E_{h},I_{h},S_{v},E_{v},I_{v}\right)={ϒ}_{v}E_{v}\left(t\right)-\Lambda_{v}I_{v}\left(t\right). \end{array}\right. \end{array}$$ (5)

Utilizing Eq (4) and considering *t* ∈ **I**, we obtain

DRLθ1L(t)=qtθ2-1Ψ(t,L(t)),0<θ1,θ2≤1,L(0)=L0. (6)

For (6), by substituting DCθ1,θ2 for DRLθ1,θ2 and making use of the Riemann-Liouville integral, we obtain:

$$\begin{array}{c} L\left(t\right)=L_{0}+\frac{\theta_{2}}{\Gamma (\theta_{1})}\int_{0}^{t}s^{\theta_{2}-1}{\left(t-s\right)}^{\theta_{1}-1}\Psi \left(s,L\left(s\right)\right)\text{ds}, \end{array}$$ (7)

where $L\left(t\right)=\{\begin{array}{c} S_{h}\left(t\right)\\ E_{h}\left(t\right)\\ I_{h}\left(t\right)\\ S_{v}\left(t\right)\\ E_{v}\left(t\right)\\ I_{v}\left(t\right) \end{array}\;L_{0}=\{\begin{array}{c} S_{h_{0}}\\ E_{h_{0}}\\ I_{h_{0}}\\ S_{v_{0}}\\ E_{v_{0}}\\ I_{v_{0}} \end{array},\;\Psi \left(t,L\left(t\right)\right)=\{\begin{array}{c} g_{1}\left(t,S_{h},E_{h},I_{h},S_{v},E_{v},I_{v}\right)\\ g_{2}\left(t,S_{h},E_{h},I_{h},S_{v},E_{v},I_{v}\right)\\ g_{3}\left(t,S_{h},E_{h},I_{h},S_{v},E_{v},I_{v}\right)\\ g_{4}\left(t,S_{h},E_{h},I_{h},S_{v},E_{v},I_{v}\right)\\ g_{5}\left(t,S_{h},E_{h},I_{h},S_{v},E_{v},I_{v}\right)\\ g_{6}\left(t,S_{h},E_{h},I_{h},S_{v},E_{v},I_{v}\right) \end{array}$.

Further, we’ll convert the problem described in Eq (1) into a fixed point problem. Consider the operator **O**, which maps from the space **V** to itself, defined as follows:

$$\begin{array}{c} O\left(L\right)\left(t\right)=L_{0}+\frac{\theta_{1}}{\Gamma (\theta_{2})}\int_{0}^{t}{\tilde{z}}^{\theta_{1}-1}{\left(t-\tilde{z}\right)}^{\theta_{2}-1}\Psi \left(\tilde{z},L\left(\tilde{z}\right)\right)d\tilde{z}. \end{array}$$ (8)

The following theorem is used in existence theory.

**Theorem 3.1** [31] *Suppose that the operator* **O**, *mapping from* **V** *to* **V**, *is completely continuous, and that the set*

F(O)={L∈V:L=αO(L),α∈[0,1]}

*is bounded. Then* **O** *has a fixed point in* V.

**Theorem 3.2**. *Suppose that* Ψ:I×V→R *is a continuous function. Then* **O** *is completely continuous*.

**proof 3.1** *Assume that* **H** *in* **V** *is bounded then there exist* **C**_Ψ_ > 0 *with* |Ψ(t, **L**(t))| ⩽ **C**_Ψ_, ∀**L** ∈ **H**. *For every* **L** ∈ **H**, *we obtain*

$$\begin{array}{cc} \|O\left(L\right)-L_{0}\| & ⩽\frac{\theta_{2}C_{\Psi }}{\Gamma (\theta_{1})}\underset{t\in I}{\text{max}}\int_{0}^{t}{\tilde{z}}^{\theta_{2}-1}{\left(t-\tilde{z}\right)}^{\theta_{2}-1}d\tilde{z},\\ & \frac{\theta_{2}C_{\Psi }}{\Gamma (\theta_{1})}\underset{t\in I}{\text{max}}\int_{0}^{1}{\tilde{z}}^{\theta_{1}-1}{\left(1-\tilde{z}\right)}^{\theta_{2}-1}t^{\theta_{1}+\theta_{2}-1}d\tilde{z}\text{,}\;\\ & ⩽\frac{\theta_{2}C_{\Psi }{\text{max}}_{t\in I}t^{\theta_{1}+\theta_{2}-1}}{\Gamma (\theta_{1})}B\left(\theta_{1},\theta_{2}\right)=\\ & \frac{\theta_{2}C_{\Psi }}{\Gamma (\theta_{1})}B\left(\theta_{1},\theta_{2}\right). \end{array}$$ (9)

*Given that* **B**(*θ*_1_, *θ*_2_) *denotes the Beta function, it follows that* **O** *is uniformly bounded*.

*Moreover, to ensure the equicontinuity of the operator* **O**, *it is required that for any t*_1_, *t*_2_ ∈ **I** *and* **L** ∈ **H**, *we have*:

$$\begin{array}{cc} \|O\left(L\right)\left(t_{1}\right)-O\left(L\right)\left(t_{2}\right)\| & ⩽\frac{\theta_{2}C}{\Gamma (\theta_{1})}\underset{t\to I}{\text{max}}\left|\int_{0}^{t_{1}}{\tilde{z}}^{\theta_{2}-1}{\left(t_{1}-\tilde{z}\right)}^{\theta_{1}-1}d\tilde{z}-\int_{0}^{t_{2}}{\tilde{z}}^{\theta_{2}-1}{\left(t_{2}-\tilde{z}\right)}^{\theta_{1}-1}d\tilde{z}\right|,\\ & ⩽\frac{\theta_{2}C_{\Psi }B\left(\theta_{1},\theta_{2}\right)}{\Gamma (\theta_{1})}\left(t_{1}^{\theta_{1}+\theta_{2}-1}-t_{2}^{\theta_{1}+\theta_{2}-1}\right)\to 0\;\;\left(t_{1}\to t_{2}\right). \end{array}$$

*Hence*, **O** *exhibits equicontinuity and therefore by the Arzelá-Ascoli theorem*, **O**(**H**) *is relatively compact and, hence*, **O** *is completely continuous*.

**Theorem 3.3** *Assuming that*, ∀ *t* ∈ **I** *and* L∈R, ∃ *a real number* **C**_Ψ_ > 0 *satisfying* |Ψ(*t*, **L**(*t*))| ⩽ **C**_Ψ_, *under these conditions, the model given in* Eq (1) *has at least one solution within the space* **V**.

**proof 3.2** *Consider* **A** = {**L** ∈ **V** : **L** = *α* **O**(**L**), *α* ∈ [0, 1]} *be a set and show that* **A** *is bounded. Let* **L** ∈ **A**, *then* **L** = *α* **O**(**L**). *For* t ∈ **I**, *we get*

$$\left\|L\right\|⩽\frac{\theta_{2}C_{\Psi }O^{\theta_{1}+\theta_{2}-1}}{\Gamma (\theta_{1})}B\left(\theta_{1},\theta_{2}\right).$$

*Hence*, **A** *is bounded. Based on Theorem 3.1 and Theorem 3.2, it can be concluded that* **O** *has a fixed point. Therefore, the model* (1) *possesses at least one solution*.

One hypothesis to explore further is the following:

(J): ∃ constant **W**_Ψ_ > 0 such that for any $L,\bar{L}\in V$, then the below inequality holds true:

$$|\Psi \left(t,L\right)-\Psi \left(t,\bar{L}\right)|⩽W_{\Psi }\left|L-\bar{L}\right|.$$

To demonstrate uniqueness, we’ll utilize Banach’s Contraction Theorem [43].

**Theorem 3.4** *If condition* (J) *is satisfied along with* Ξ < 1, *where*

$$\begin{array}{c} \Xi =\frac{\theta_{2}W_{\Psi }O^{\theta_{1}+\theta_{2}-1}}{\Gamma (\theta_{2})}B\left(\theta_{1},\theta_{2}\right) \end{array}$$ (10)

*then the solution to the provided model* (1) *is unique*.

**proof 3.3** *Consider* max_*t*∈**I**_ |Ψ(t, 0)| = **G**_Ψ_ < ∞, *and take h such that*

$$h⩾\frac{\theta_{2}O^{\theta_{1}+\theta_{2}-1}B\left(\theta_{1},\theta_{2}\right)G_{\Psi }}{\Gamma \left(\theta_{1}\right)-\theta_{2}O^{\theta_{1}+\theta_{2}-1}B\left(\theta_{1},\theta_{2}\right)W_{\Psi }}$$

*We demonstrate that* $O(A_{h})\subset A_{h}$, *where* $A_{h}=\left\{L\in V:\|L\|⩽h\right\}$. *For* $L\in A_{h}$, *we have*

$$\begin{array}{cc} \left\|O\left(L\right)\right\| & ⩽\frac{\theta_{2}}{\Gamma (\theta_{1})}\underset{t\in I}{\text{max}}\int_{0}^{t}{\tilde{z}}^{\theta_{2}-1}{\left(t-s\right)}^{\theta_{1}-1}\left(|\Psi \left(t,0\right)|+|\Psi \left(t,L\left(t\right)\right)-\Psi \left(t,0\right)|\right)d\tilde{z},\\ & ⩽\frac{\theta_{2}O^{\theta_{1}+\theta_{2}-1}B\left(\theta_{1},\theta_{2}\right)(W_{\Psi }\left\|L\right\|+G_{\Psi })}{\Gamma (\theta_{1})}.\\ & ⩽\frac{\theta_{2}O^{\theta_{1}+\theta_{2}-1}B\left(\theta_{1},\theta_{2}\right)\left(W_{\Psi }\right)h+G_{\Psi }}{\Gamma (\theta_{1})}.\\ & ⩽h. \end{array}$$

*Consider the function* **O** : **V** → **V** *defined by* Eq (8). *Given condition* (J), *for all* t ∈ **I** *and for any* $L,\bar{L}\in V$, *we obtain*:

$$\begin{array}{cc} \|O\left(L\right)-O\left(\bar{L}\right)\| & ⩽\frac{\theta_{2}}{\Gamma (\theta_{1})}\underset{t\in I}{\text{max}}|\int_{0}^{t}{\tilde{z}}^{\theta_{2}-1}{\left(t-\tilde{z}\right)}^{\theta_{1}-1}\Psi \left(\tilde{z},L\left(\tilde{z}\right)\right)d\tilde{z}\\ & -\int_{0}^{t}{\tilde{z}}^{\theta_{2}-1}{\left(t-\tilde{z}\right)}^{\theta_{1}-1}\Psi \left(\tilde{z},\bar{L}\left(\tilde{z}\right)\right)d\tilde{z}|,\\ & ⩽\Xi \|L-\bar{L}\|. \end{array}$$ (11)

*Hence, it follows that* **O** *fulfills the contraction condition expressed as in* (11). *Therefore, the integral* Eq (7) *admits a unique solution, which also holds true for model* (1).

## 4 Ulam stability

In this section, we will examine the stability of the model described in (1) by introducing a minor perturbation denoted as Π ∈ *C*(**I**). This perturbation solely relies on the solution, by taking the constraint Π(0) = 0. Next,

- for *ϵ* > 0, |Π(t)| ⩽ *ϵ*,
-
DFFPθ1,θ2L(t)=Ψ(t,L(t))+Π(t) .

**Lemma 4.1** [31] *The perturbed problem’s solution will be*:

DFFPθ1,θ2L(t)=Ψ(t,L(t))+Π(t)L(0)=L0

*satisfies*

$$\begin{array}{c} |L\left(t\right)-\left(L_{0}+\frac{\theta_{2}}{\Gamma (\theta_{1})}\int_{0}^{t}{\tilde{z}}^{\theta_{2}-1}{\left(t-\tilde{z}\right)}^{\theta_{1}-1}\Psi \left(\tilde{z},L\left(\tilde{z}\right)\right)d\tilde{z}\right)|⩽(\frac{\theta_{2}O^{\theta_{1}+\theta_{2}-1}}{\Gamma (\theta_{1})}B\left(\theta_{1},\theta_{2}\right))\epsilon =C_{\theta_{1},\theta_{2}}\epsilon . \end{array}$$ (12)

**Theorem 4.1** *Taking into account assumptions* H *and* Eq (12) *in Lemma 4.1, we can conclude that the solution to* Eq (7) *demonstrates UHS. Consequently, Hence, the entire system achieves UHS, when* Ξ (*as defined in* (10)) < 1.

**proof 4.1** *Considering a unique solution* **Z** ∈ **V** *and another solution* **L** ∈ **V** *of* Eq (7), *we have*

$$\begin{array}{cc} \left|L\left(t\right)-Z\left(t\right)\right| & =|L\left(t\right)-\left(Z_{0}+\frac{\theta_{2}}{\Gamma (\theta_{1})}\int_{0}^{t}{\tilde{z}}^{\theta_{2}-1}{\left(t-\tilde{z}\right)}^{\theta_{1}-1}\Psi \left(\tilde{z},Z\left(\tilde{z}\right)\right)d\tilde{z}\right)|\\ & ⩽|L\left(t\right)-\left(L_{0}+\frac{\theta_{2}}{\Gamma (\theta_{1})}\int_{0}^{t}{\tilde{z}}^{\theta_{2}-1}{\left(t-\tilde{z}\right)}^{\theta_{1}-1}\Psi \left(\tilde{z},L\left(\tilde{z}\right)\right)d\tilde{z}\right)\\ & +\left(L_{0}+\frac{\theta_{2}}{\Gamma (\theta_{1})}\int_{0}^{t}{\tilde{z}}^{\theta_{2}-1}{\left(t-\tilde{z}\right)}^{\theta_{1}-1}\Psi \left(\tilde{z},L\left(\tilde{z}\right)\right)d\tilde{z}\right)\\ & -\left(Z_{0}+\frac{\theta_{2}}{\Gamma (\theta_{1})}\int_{0}^{t}{\tilde{z}}^{\theta_{2}-1}{\left(t-\tilde{z}\right)}^{\theta_{1}-1}\Psi \left(\tilde{z},Z\left(\tilde{z}\right)\right)d\tilde{z}\right)\left|)\right|\\ & ⩽C_{\theta_{1},\theta_{2}}\epsilon +\frac{\theta_{2}WO^{\theta_{1}+\theta_{2}-1}B\left(\theta_{1},\theta_{2}\right)}{\Gamma (\theta_{1})}\|L-Z\|. \end{array}$$

*Now*

$$\begin{array}{c} \left\|L-Z\right\|⩽C_{\theta_{1},\theta_{2}}\epsilon +\Xi \left\|L-Z\right\|. \end{array}$$ (13)

*From* (13), *we get*

$$\begin{array}{c} \left\|L-Z\right\|⩽(\frac{C_{\theta_{1},\theta_{2}}}{1-\Xi })\epsilon . \end{array}$$ (14)

*Hence, the implication derived from* Eq (14) *suggests that the solution to* Eq (7) *displays UHS. Consequently, we can infer that the solution to the given problem also possesses UHS*.

## 5 Numerical scheme of FF derivative

Here, we will develop a numerical algorithm for simulating the suggested model. Specifically, we will outline the steps for constructing Eq (7) of the model using numerical methods.

$$\begin{array}{c} \left\{ \begin{array}{c} S_{h}=S_{h_{0}}\frac{\theta_{2}}{\Gamma \left(\theta_{1}\right)}\int_{0}^{t}{\tilde{\tilde{z}}}^{\theta_{2}-1}{\left(t-\tilde{z}\right)}^{\theta_{1}-1}g_{1}\left(\tilde{z},S_{h}\left(\tilde{z}\right),E_{h}\left(\tilde{z}\right),I_{h}\left(\tilde{z}\right),S_{v}\left(\tilde{z}\right),E_{v}\left(\tilde{z}\right),I_{v}\left(\tilde{z}\right)\right)d\tilde{z},\\ E_{h}=E_{h_{0}}\frac{\theta_{2}}{\Gamma \left(\theta_{1}\right)}\int_{0}^{t}{\tilde{z}}^{\theta_{2}-1}{\left(t-\tilde{z}\right)}^{\theta_{1}-1}g_{2}\left(\tilde{z},S_{h}\left(\tilde{z}\right),E_{h}\left(\tilde{z}\right),I_{h}\left(\tilde{z}\right),S_{v}\left(\tilde{z}\right),E_{v}\left(\tilde{z}\right),I_{v}\left(\tilde{z}\right)\right)d\tilde{z},\\ I_{h}=I_{h_{0}}\frac{\theta_{2}}{\Gamma \left(\theta_{1}\right)}\int_{0}^{t}{\tilde{z}}^{\theta_{2}-1}{\left(t-\tilde{z}\right)}^{\theta_{1}-1}g_{3}\left(\tilde{z},S_{h}\left(\tilde{z}\right),E_{h}\left(\tilde{z}\right),I_{h}\left(\tilde{z}\right),S_{v}\left(\tilde{z}\right),E_{v}\left(\tilde{z}\right),I_{v}\left(\tilde{z}\right)\right)d\tilde{z},\\ S_{v}=S_{v_{0}}\frac{\theta_{2}}{\Gamma \left(\theta_{1}\right)}\int_{0}^{t}{\tilde{z}}^{\theta_{2}-1}{\left(t-\tilde{z}\right)}^{\theta_{1}-1}g_{4}\left(\tilde{z},S_{h}\left(\tilde{z}\right),E_{h}\left(\tilde{z}\right),I_{h}\left(\tilde{z}\right),S_{v}\left(\tilde{z}\right),E_{v}\left(\tilde{z}\right),I_{v}\left(\tilde{z}\right)\right)d\tilde{z},\\ E_{v}=E_{v_{0}}\frac{\theta_{2}}{\Gamma \left(\theta_{1}\right)}\int_{0}^{t}{\tilde{z}}^{\theta_{2}-1}{\left(t-\tilde{z}\right)}^{\theta_{1}-1}g_{5}\left(\tilde{z},S_{h}\left(\tilde{z}\right),E_{h}\left(\tilde{z}\right),I_{h}\left(\tilde{z}\right),S_{v}\left(\tilde{z}\right),E_{v}\left(\tilde{z}\right),I_{v}\left(\tilde{z}\right)\right)d\tilde{z},\\ I_{v}=I_{v_{0}}\frac{\theta_{2}}{\Gamma \left(\theta_{1}\right)}\int_{0}^{t}{\tilde{z}}^{\theta_{2}-1}{\left(t-\tilde{z}\right)}^{\theta_{1}-1}g_{6}\left(\tilde{z},S_{h}\left(\tilde{z}\right),E_{h}\left(\tilde{z}\right),I_{h}\left(\tilde{z}\right),S_{v}\left(\tilde{z}\right),E_{v}\left(\tilde{z}\right),I_{v}\left(\tilde{z}\right)\right)d\tilde{z}. \end{array}\right. \end{array}$$ (15)

Here, we introduce a novel approach to the numerical method for this system when considering the time step *t*_*m*+1_. This results in the following system transformation:

$$\begin{array}{c} \left\{ \begin{array}{c} S_{h_{m+1}}=S_{h_{0}}+\frac{\theta_{2}}{\Gamma \left(\theta_{1}\right)}\int_{0}^{t_{m+1}}{\tilde{z}}^{\theta_{2}-1}{\left(t_{m+1}-\tilde{z}\right)}^{\theta_{1}-1}g_{1}\left(\tilde{z},S_{h}\left(\tilde{z}\right),E_{h}\left(\tilde{z}\right),I_{h}\left(\tilde{z}\right),S_{v}\left(\tilde{z}\right),E_{v}\left(\tilde{z}\right),I_{v}\left(\tilde{z}\right)\right)d\tilde{z},\\ E_{h_{m+1}}=S_{h_{0}}+\frac{\theta_{2}}{\Gamma \left(\theta_{1}\right)}\int_{0}^{t_{m+1}}{\tilde{z}}^{\theta_{2}-1}{\left(t_{m+1}-\tilde{z}\right)}^{\theta_{1}-1}g_{2}\left(\tilde{z},S_{h}\left(\tilde{z}\right),E_{h}\left(\tilde{z}\right),I_{h}\left(\tilde{z}\right),S_{v}\left(\tilde{z}\right),E_{v}\left(\tilde{z}\right),I_{v}\left(\tilde{z}\right)\right)d\tilde{z},\\ I_{h_{m+1}}=I_{h_{0}}+\frac{\theta_{2}}{\Gamma \left(\theta_{1}\right)}\int_{0}^{t_{m+1}}{\tilde{z}}^{\theta_{2}-1}{\left(t_{m+1}-\tilde{z}\right)}^{\theta_{1}-1}g_{3}\left(\tilde{z},S_{h}\left(\tilde{z}\right),E_{h}\left(\tilde{z}\right),I_{h}\left(\tilde{z}\right),S_{v}\left(\tilde{z}\right),E_{v}\left(\tilde{z}\right),I_{v}\left(\tilde{z}\right)\right)d\tilde{z},\\ S_{v_{m+1}}=S_{v_{0}}+\frac{\theta_{2}}{\Gamma \left(\theta_{1}\right)}\int_{0}^{t_{m+1}}{\tilde{z}}^{\theta_{2}-1}{\left(t_{m+1}-\tilde{z}\right)}^{\theta_{1}-1}g_{4}\left(\tilde{z},S_{h}\left(\tilde{z}\right),E_{h}\left(\tilde{z}\right),I_{h}\left(\tilde{z}\right),S_{v}\left(\tilde{z}\right),E_{v}\left(\tilde{z}\right),I_{v}\left(\tilde{z}\right)\right)d\tilde{z},\\ E_{v_{m+1}}=E_{v_{0}}+\frac{\theta_{2}}{\Gamma \left(\theta_{1}\right)}\int_{0}^{t_{m+1}}{\tilde{z}}^{\theta_{2}-1}{\left(t_{m+1}-\tilde{z}\right)}^{\theta_{1}-1}g_{5}\left(\tilde{z},S_{h}\left(\tilde{z}\right),E_{h}\left(\tilde{z}\right),I_{h}\left(\tilde{z}\right),S_{v}\left(\tilde{z}\right),E_{v}\left(\tilde{z}\right),I_{v}\left(\tilde{z}\right)\right)d\tilde{z},\\ I_{v_{m+1}}=I_{v_{0}}+\frac{\theta_{2}}{\Gamma \left(\theta_{1}\right)}\int_{0}^{t_{m+1}}{\tilde{z}}^{\theta_{2}-1}{\left(t_{m+1}-\tilde{z}\right)}^{\theta_{1}-1}g_{6}\left(\tilde{z},S_{h}\left(\tilde{z}\right),E_{h}\left(\tilde{z}\right),I_{h}\left(\tilde{z}\right),S_{v}\left(\tilde{z}\right),E_{v}\left(\tilde{z}\right),I_{v}\left(\tilde{z}\right)\right)d\tilde{z}. \end{array}\right. \end{array}$$ (16)

Next, we proceed to estimate the integrals derived in (16)

$$\begin{array}{c} \left\{ \begin{array}{c} S_{h_{m+1}}=S_{h_{0}}+\frac{\theta_{2}}{\Gamma \left(\theta_{1}\right)}\sum_{n=0}^{m}\int_{t_{n}}^{t_{n+1}}{\tilde{z}}^{\theta_{2}-1}{\left(t_{m+1}-\tilde{z}\right)}^{\theta_{1}-1}g_{1}\left(\tilde{z},S_{h}\left(\tilde{z}\right),E_{h}\left(\tilde{z}\right),I_{h}\left(\tilde{z}\right),S_{v}\left(\tilde{z}\right),E_{v}\left(\tilde{z}\right),I_{v}\left(\tilde{z}\right)\right)d\tilde{z},\\ E_{h_{m+1}}=E_{h_{0}}+\frac{\theta_{2}}{\Gamma \left(\theta_{1}\right)}\sum_{n=0}^{m}\int_{t_{n}}^{t_{n+1}}{\tilde{z}}^{\theta_{2}-1}{\left(t_{m+1}-\tilde{z}\right)}^{\theta_{1}-1}g_{2}\left(\tilde{z},S_{h}\left(\tilde{z}\right),E_{h}\left(\tilde{z}\right),I_{h}\left(\tilde{z}\right),S_{v}\left(\tilde{z}\right),E_{v}\left(\tilde{z}\right),I_{v}\left(\tilde{z}\right)\right)d\tilde{z},\\ I_{h_{m+1}}=I_{h_{0}}+\frac{\theta_{2}}{\Gamma \left(\theta_{1}\right)}\sum_{n=0}^{m}\int_{t_{n}}^{t_{n+1}}{\tilde{z}}^{\theta_{2}-1}{\left(t_{m+1}-\tilde{z}\right)}^{\theta_{1}-1}g_{3}\left(\tilde{z},S_{h}\left(\tilde{z}\right),E_{h}\left(\tilde{z}\right),I_{h}\left(\tilde{z}\right),S_{v}\left(\tilde{z}\right),E_{v}\left(\tilde{z}\right),I_{v}\left(\tilde{z}\right)\right)d\tilde{z},\\ S_{v_{m+1}}=S_{v_{0}}+\frac{\theta_{2}}{\Gamma \left(\theta_{1}\right)}\sum_{n=0}^{m}\int_{t_{n}}^{t_{n+1}}{\tilde{z}}^{\theta_{2}-1}{\left(t_{m+1}-\tilde{z}\right)}^{\theta_{1}-1}g_{4}\left(\tilde{z},S_{h}\left(\tilde{z}\right),E_{h}\left(\tilde{z}\right),I_{h}\left(\tilde{z}\right),S_{v}\left(\tilde{z}\right),E_{v}\left(\tilde{z}\right),I_{v}\left(\tilde{z}\right)\right)d\tilde{z},\\ E_{v_{m+1}}=E_{v_{0}}+\frac{\theta_{2}}{\Gamma \left(\theta_{1}\right)}\sum_{n=0}^{m}\int_{t_{n}}^{t_{n+1}}{\tilde{z}}^{\theta_{2}-1}{\left(t_{m+1}-\tilde{z}\right)}^{\theta_{1}-1}g_{5}\left(\tilde{z},S_{h}\left(\tilde{z}\right),E_{h}\left(\tilde{z}\right),I_{h}\left(\tilde{z}\right),S_{v}\left(\tilde{z}\right),E_{v}\left(\tilde{z}\right),I_{v}\left(\tilde{z}\right)\right)d\tilde{z},\\ I_{v_{m+1}}=I_{v_{0}}+\frac{\theta_{2}}{\Gamma \left(\theta_{1}\right)}\sum_{n=0}^{m}\int_{t_{n}}^{t_{n+1}}{\tilde{z}}^{\theta_{2}-1}{\left(t_{m+1}-\tilde{z}\right)}^{\theta_{1}-1}g_{6}\left(\tilde{z},S_{h}\left(\tilde{z}\right),E_{h}\left(\tilde{z}\right),I_{h}\left(\tilde{z}\right),S_{v}\left(\tilde{z}\right),E_{v}\left(\tilde{z}\right),I_{v}\left(\tilde{z}\right)\right)d\tilde{z}, \end{array}\right. \end{array}$$ (17)

In the restricted time interval denoted as [t_m_, t_m+1_], we employ a Lagrangian piece-wise interpolation method to approximate the function ${\tilde{z}}^{\theta_{2}-1}g_{j}\left(\tilde{z},S_{h},E_{h},I_{h},S_{v},E_{v},I_{v}\right)$, where *j* ranges from 1 to 6. This approximation is carried out over a subinterval *l* = t_n_ − t_n−1_.

$$\begin{array}{c} S_{h_{n}}^{⋆}\approx \frac{1}{l}[(t-t_{n-1})t_{n}^{\theta_{2}-1}g_{1}\left(t_{n},S_{h_{n}},E_{h_{n}},I_{h_{n}},S_{v_{n}},E_{v_{n}},I_{v_{n}}\right)\\ -(t-t_{n})t_{n-1}^{\theta_{2}-1}g_{1}\left(t_{n-1},S_{h_{n-1}},E_{h_{n-1}},I_{h_{n-1}},S_{v_{n-1}},E_{v_{n-1}},I_{v_{n-1}}\right)],\\ E_{h_{n}}^{⋆}\approx \frac{1}{l}[(t-t_{n-1})t_{n}^{\theta_{2}-1}g_{2}\left(t_{n},S_{h_{n}},E_{h_{n}},I_{h_{n}},S_{v_{n}},E_{v_{n}},I_{v_{n}}\right)\\ -(t-t_{n})t_{n-1}^{\theta_{2}-1}g_{2}\left(t_{n-1},S_{h_{n-1}},E_{h_{n-1}},I_{h_{n-1}},S_{v_{n-1}},E_{v_{n-1}},I_{v_{n-1}}\right)],\\ I_{h_{n}}^{⋆}\approx \frac{1}{l}[(t-t_{n-1})t_{n}^{\theta_{2}-1}g_{3}\left(t_{n},S_{h_{n}},E_{h_{n}},I_{h_{n}},S_{v_{n}},E_{v_{n}},I_{v_{n}}\right)\\ -(t-t_{n})t_{n-1}^{\theta_{2}-1}g_{3}\left(t_{n-1},S_{h_{n-1}},E_{h_{n-1}},I_{h_{n-1}},S_{v_{n-1}},E_{v_{n-1}},I_{v_{n-1}}\right)],\\ S_{v_{n}}^{⋆}\approx \frac{1}{l}[(t-t_{n-1})t_{n}^{\theta_{2}-1}g_{4}\left(t_{n},S_{h_{n}},E_{h_{n}},I_{h_{n}},S_{v_{n}},E_{v_{n}},I_{v_{n}}\right)\\ -(t-t_{n})t_{n-1}^{\theta_{2}-1}g_{4}\left(t_{n-1},S_{h_{n-1}},E_{h_{n-1}},I_{h_{n-1}},S_{v_{n-1}},E_{v_{n-1}},I_{v_{n-1}}\right)],\\ E_{v_{n}}^{⋆}\approx \frac{1}{l}[(t-t_{n-1})t_{n}^{\theta_{2}-1}g_{5}\left(t_{n},S_{h_{n}},E_{h_{n}},I_{h_{n}},S_{v_{n}},E_{v_{n}},I_{v_{n}}\right)\\ -(t-t_{n})t_{n-1}^{\theta_{2}-1}g_{5}\left(t_{n-1},S_{h_{n-1}},E_{h_{n-1}},I_{h_{n-1}},S_{v_{n-1}},E_{v_{n-1}},I_{v_{n-1}}\right)],\\ I_{v_{n}}^{⋆}\approx \frac{1}{l}[(t-t_{n-1})t_{n}^{\theta_{2}-1}g_{6}\left(t_{n},S_{h_{n}},E_{h_{n}},I_{h_{n}},S_{v_{n}},E_{v_{n}},I_{v_{n}}\right)\\ -(t-t_{n})t_{n-1}^{\theta_{2}-1}g_{6}\left(t_{n-1},S_{h_{n-1}},E_{h_{n-1}},I_{h_{n-1}},S_{v_{n-1}},E_{v_{n-1}},I_{v_{n-1}}\right)]. \end{array}$$ (18)

Using (18) in (17), we get

$$\begin{array}{c} \left\{ \begin{array}{c} S_{h_{m+1}}=S_{0}+\frac{\theta_{2}}{\Gamma \left(\theta_{1}\right)}\sum_{n=0}^{m}\int_{t_{n}}^{t_{n+1}}\;{\tilde{z}}^{\theta_{2}-1}{\left(t_{m+1}-\tilde{z}\right)}^{\theta_{1}-1}S_{h_{n}}^{⋆}\left(\tilde{z}\right)d\tilde{z},\\ E_{h_{m+1}}=E_{0}+\frac{\theta_{2}}{\Gamma \left(\theta_{1}\right)}\sum_{n=0}^{m}\int_{t_{n}}^{t_{n+1}}\;{\tilde{z}}^{\theta_{2}-1}{\left(t_{m+1}-\tilde{z}\right)}^{\theta_{1}-1}E_{h_{n}}^{⋆}\left(\tilde{z}\right)d\tilde{z},\\ I_{h_{m+1}}=I_{0}+\frac{\theta_{2}}{\Gamma \left(\theta_{1}\right)}\sum_{n=0}^{m}\int_{t_{n}}^{t_{n+1}}\;{\tilde{z}}^{\theta_{2}-1}{\left(t_{m+1}-\tilde{z}\right)}^{\theta_{1}-1}I_{h_{n}}^{⋆}\left(\tilde{z}\right)d\tilde{z},\\ S_{v_{m+1}}=S_{0}+\frac{\theta_{2}}{\Gamma \left(\theta_{1}\right)}\sum_{n=0}^{m}\int_{t_{n}}^{t_{n+1}}\;{\tilde{z}}^{\theta_{2}-1}{\left(t_{m+1}-\tilde{z}\right)}^{\theta_{1}-1}S_{h_{v}}^{⋆}\left(\tilde{z}\right)d\tilde{z},\\ E_{v_{m+1}}=E_{0}+\frac{\theta_{2}}{\Gamma \left(\theta_{1}\right)}\sum_{n=0}^{m}\int_{t_{n}}^{t_{n+1}}\;{\tilde{z}}^{\theta_{2}-1}{\left(t_{m+1}-\tilde{z}\right)}^{\theta_{1}-1}E_{v_{n}}^{⋆}\left(\tilde{z}\right)d\tilde{z},\\ I_{v_{m+1}}=I_{0}+\frac{\theta_{2}}{\Gamma \left(\theta_{1}\right)}\sum_{n=0}^{m}\int_{t_{n}}^{t_{n+1}}\;{\tilde{z}}^{\theta_{2}-1}{\left(t_{m+1}-\tilde{z}\right)}^{\theta_{1}-1}I_{v_{n}}^{⋆}\left(\tilde{z}\right)d\tilde{z}, \end{array}\right. \end{array}$$ (19)

By simplifying the (19), we get the numerical method of model (1) using FF derivatives in Caputo sense

$$\begin{array}{c} S_{h_{m+1}}=S_{0}+\frac{\theta_{2}l^{\theta_{1}}}{\Gamma (\theta_{1}+2)}\sum_{n=0}^{m}[t_{n}^{\theta_{2}-1}g_{1}\left(t_{n},S_{h_{n}},E_{h_{n}},I_{h_{n}},S_{v_{n}},E_{v_{n}},I_{v_{n}}\right)(\left(m+2+\theta_{1}-n\right){\left(m+1-n\right)}^{\theta_{1}}\\ -\left(m+2+2\theta_{1}-n\right){\left(m-n\right)}^{\theta_{1}})-t_{n-1}^{\theta_{2}-1}g_{1}\left(t_{n},S_{h_{n-1}},E_{h_{n-1}},I_{h_{n-1}},S_{v_{n-1}},E_{v_{n-1}},I_{v_{n-1}}\right)\\ \times ({\left(m+1-n\right)}^{\theta_{1}+1}-\left(m-n+1+\theta_{1}\right){\left(m-n\right)}^{\theta_{1}})], \end{array}$$

$$\begin{array}{c} E_{h_{m+1}}=E_{0}+\frac{\theta_{2}l^{\theta_{1}}}{\Gamma (\theta_{1}+2)}\sum_{n=0}^{m}[t_{n}^{\theta_{2}-1}g_{2}\left(t_{n},S_{h_{n}},E_{h_{n}},I_{h_{n}},S_{v_{n}},E_{v_{n}},I_{v_{n}}\right)(\left(m+2+\theta_{1}-n\right){\left(m+1-n\right)}^{\theta_{1}}\\ -\left(m+2+2\theta_{1}-n\right){\left(m-n\right)}^{\theta_{1}})-t_{n-1}^{\theta_{2}-1}g_{2}\left(t_{n},S_{h_{n-1}},E_{h_{n-1}},I_{h_{n-1}},S_{v_{n-1}},E_{v_{n-1}},I_{v_{n-1}}\right)\\ \times ({\left(m+1-n\right)}^{\theta_{1}+1}-\left(m-n+1+\theta_{1}\right){\left(m-n\right)}^{\theta_{1}})], \end{array}$$

$$\begin{array}{c} I_{h_{m+1}}=I_{0}+\frac{\theta_{2}l^{\theta_{1}}}{\Gamma (\theta_{1}+2)}\sum_{n=0}^{m}[t_{n}^{\theta_{2}-1}g_{3}\left(t_{n},S_{h_{n}},E_{h_{n}},I_{h_{n}},S_{v_{n}},E_{v_{n}},I_{v_{n}}\right)(\left(m+2+\theta_{1}-n\right){\left(m+1-n\right)}^{\theta_{1}}\\ -\left(m+2+2\theta_{1}-n\right){\left(m-n\right)}^{\theta_{1}})-t_{n-1}^{\theta_{2}-1}g_{3}\left(t_{n},S_{h_{n-1}},E_{h_{n-1}},I_{h_{n-1}},S_{v_{n-1}},E_{v_{n-1}},I_{v_{n-1}}\right)\\ \times ({\left(m+1-n\right)}^{\theta_{1}+1}-\left(m-n+1+\theta_{1}\right){\left(m-n\right)}^{\theta_{1}})], \end{array}$$

$$\begin{array}{c} S_{v_{m+1}}=S_{0}+\frac{\theta_{2}l^{\theta_{1}}}{\Gamma (\theta_{1}+2)}\sum_{n=0}^{m}[t_{n}^{\theta_{2}-1}g_{4}\left(t_{n},S_{h_{n}},E_{h_{n}},I_{h_{n}},S_{v_{n}},E_{v_{n}},I_{v_{n}}\right)(\left(m+2+\theta_{1}-n\right){\left(m+1-n\right)}^{\theta_{1}}\\ -\left(m+2+2\theta_{1}-n\right){\left(m-n\right)}^{\theta_{1}})-t_{n-1}^{\theta_{2}-1}g_{4}\left(t_{n},S_{h_{n-1}},E_{h_{n-1}},I_{h_{n-1}},S_{v_{n-1}},E_{v_{n-1}},I_{v_{n-1}}\right)\\ \times ({\left(m+1-n\right)}^{\theta_{1}+1}-\left(m-n+1+\theta_{1}\right){\left(m-n\right)}^{\theta_{1}})], \end{array}$$

$$\begin{array}{c} E_{v_{m+1}}=E_{0}+\frac{\theta_{2}l^{\theta_{1}}}{\Gamma (\theta_{1}+2)}\sum_{n=0}^{m}[t_{n}^{\theta_{2}-1}g_{5}\left(t_{n},S_{h_{n}},E_{h_{n}},I_{h_{n}},S_{v_{n}},E_{v_{n}},I_{v_{n}}\right)(\left(m+2+\theta_{1}-n\right){\left(m+1-n\right)}^{\theta_{1}}\\ -\left(m+2+2\theta_{1}-n\right){\left(m-n\right)}^{\theta_{1}})-t_{n-1}^{\theta_{2}-1}g_{5}\left(t_{n},S_{h_{n-1}},E_{h_{n-1}},I_{h_{n-1}},S_{v_{n-1}},E_{v_{n-1}},I_{v_{n-1}}\right)\\ \times ({\left(m+1-n\right)}^{\theta_{1}+1}-\left(m-n+1+\theta_{1}\right){\left(m-n\right)}^{\theta_{1}})], \end{array}$$

$$\begin{array}{c} I_{v_{m+1}}=I_{0}+\frac{\theta_{2}l^{\theta_{1}}}{\Gamma (\theta_{1}+2)}\sum_{n=0}^{m}[t_{n}^{\theta_{2}-1}g_{6}\left(t_{n},S_{h_{n}},E_{h_{n}},I_{h_{n}},S_{v_{n}},E_{v_{n}},I_{v_{n}}\right)(\left(m+2+\theta_{1}-n\right){\left(m+1-n\right)}^{\theta_{1}}\\ -\left(m+2+2\theta_{1}-n\right){\left(m-n\right)}^{\theta_{1}})-t_{n-1}^{\theta_{2}-1}g_{6}\left(t_{n},S_{h_{n-1}},E_{h_{n-1}},I_{h_{n-1}},S_{v_{n-1}},E_{v_{n-1}},I_{v_{n-1}}\right)\\ \times ({\left(m+1-n\right)}^{\theta_{1}+1}-\left(m-n+1+\theta_{1}\right){\left(m-n\right)}^{\theta_{1}})]. \end{array}$$

## 6 Numerical simulation and discussion

**Fig 2:**
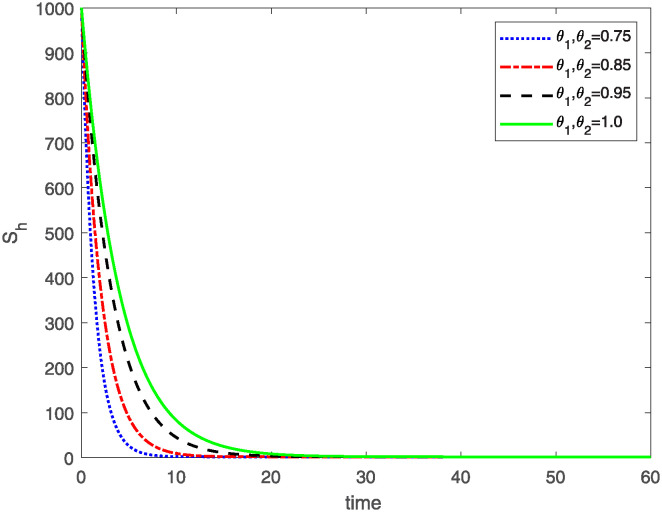
$S_{h}\left(t\right)$ class approximate solution of model (4) for same values of FO *θ*_1_ and FD *θ*_2_.

**Fig 3:**
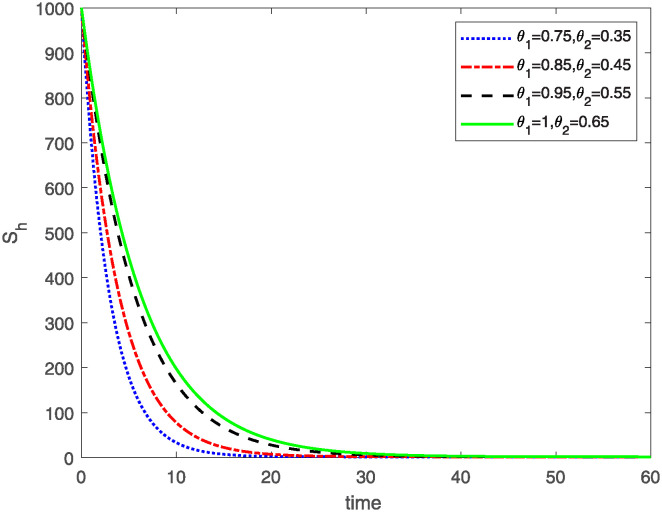
$S_{h}\left(t\right)$ class approximate solution for different values of *θ*_1_*θ*_2_.

**Fig 4:**
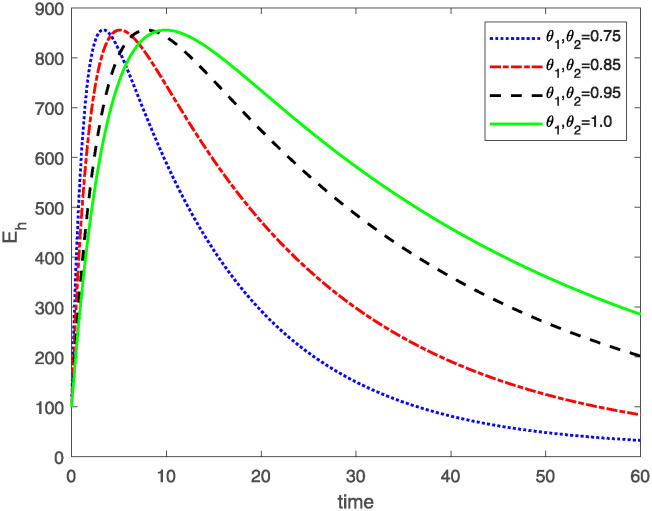
$E_{h}\left(t\right)$ class approximate solution of model (4) for same values of FO *θ*_1_ and FD *θ*_2_.

**Fig 5:**
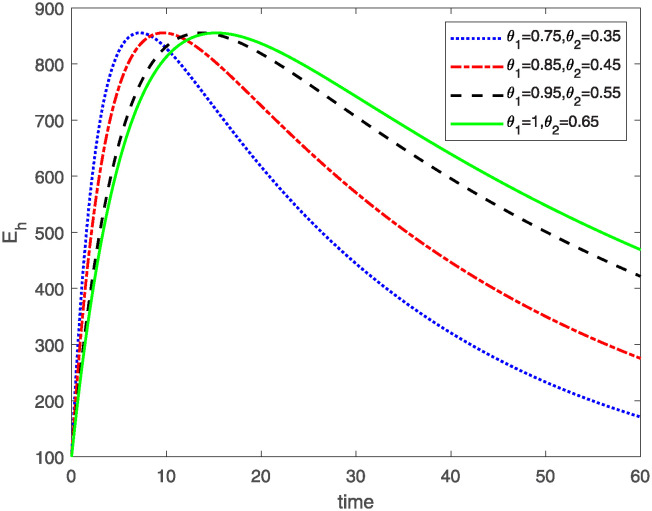
Plot for class $E_{h}\left(t\right)$ of model (4) for different values of FO *θ*_1_ and FD *θ*_2_.

**Fig 6:**
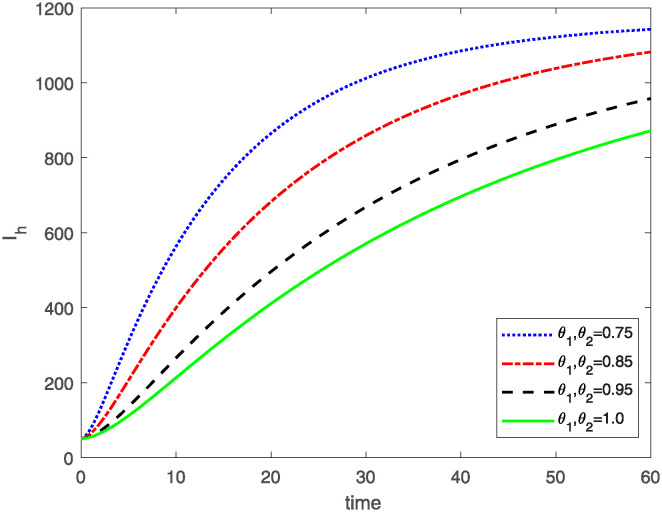
Plot for class $I_{h}\left(t\right)$ for same values of FO *θ*_1_ and FD *θ*_2_.

**Fig 7:**
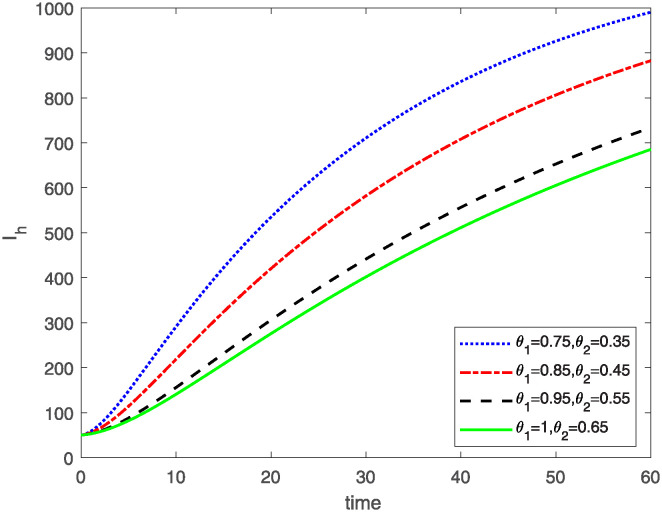
Plot for class $I_{h}\left(t\right)$ for different values of FO *θ*_1_ and FD *θ*_2_.

**Fig 8:**
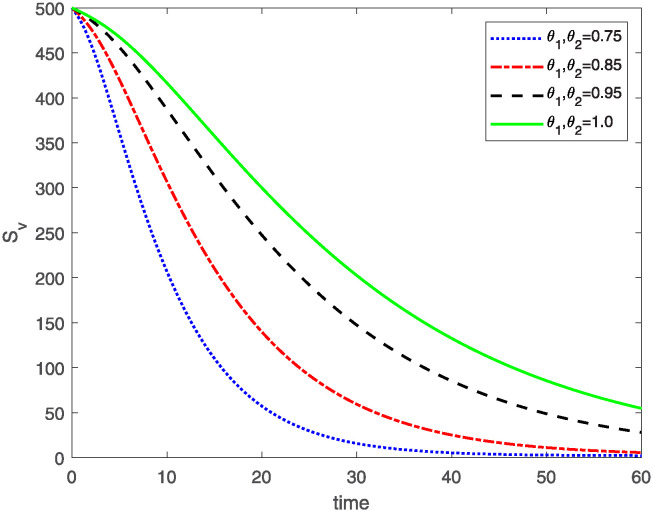
Plot of $S_{v}\left(t\right)$ for same values of FO *θ*_1_ and FD *θ*_2_.

**Fig 9:**
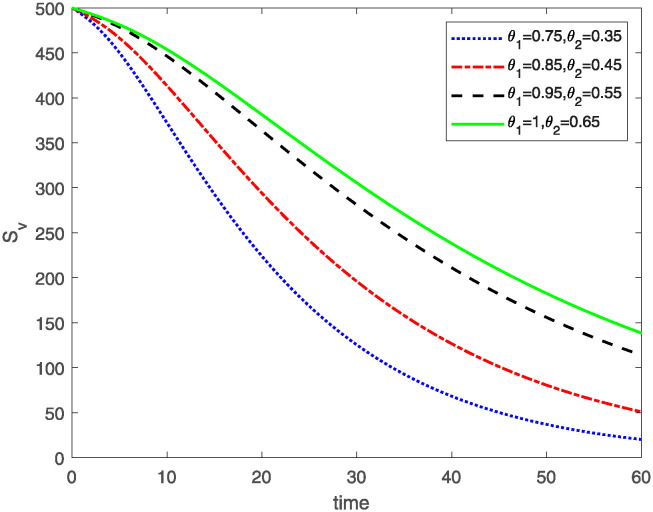
Plot of $S_{v}\left(t\right)$ for different values of FO *θ*_1_ and FD *θ*_2_.

**Fig 10:**
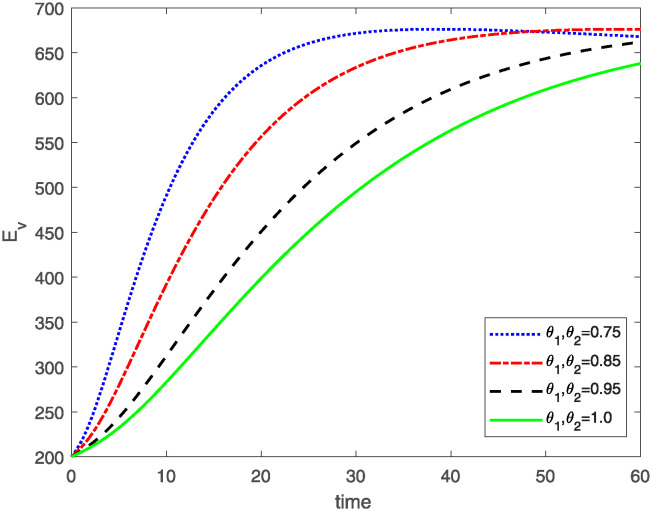
Plot for class $E_{v}\left(t\right)$ for same values of *θ*_1_*θ*_2_.

**Fig 11:**
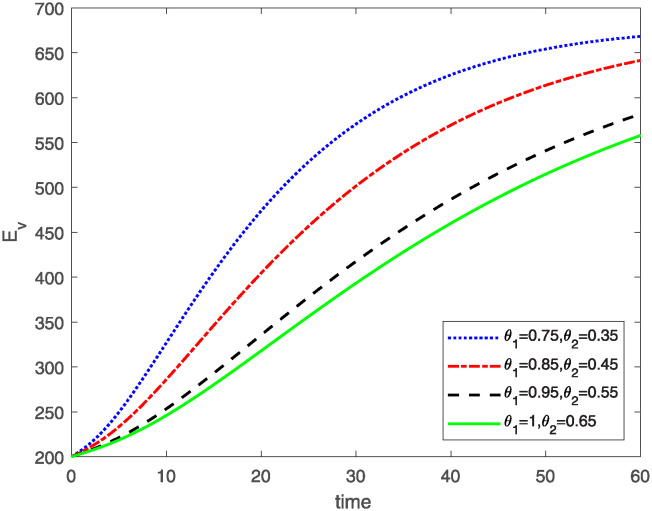
Plot for class $E_{v}\left(t\right)$ for different values of *θ*_1_*θ*_2_.

**Fig 12:**
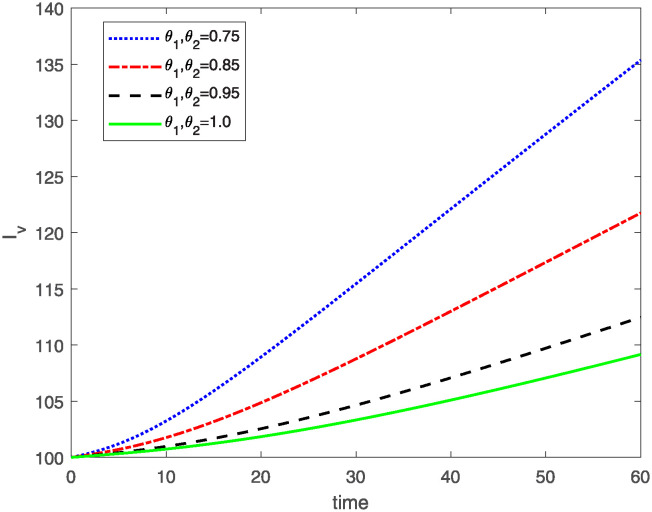
Plot for class $I_{v}\left(t\right)$ for same values of *θ*_1_*θ*_2_.

**Fig 13:**
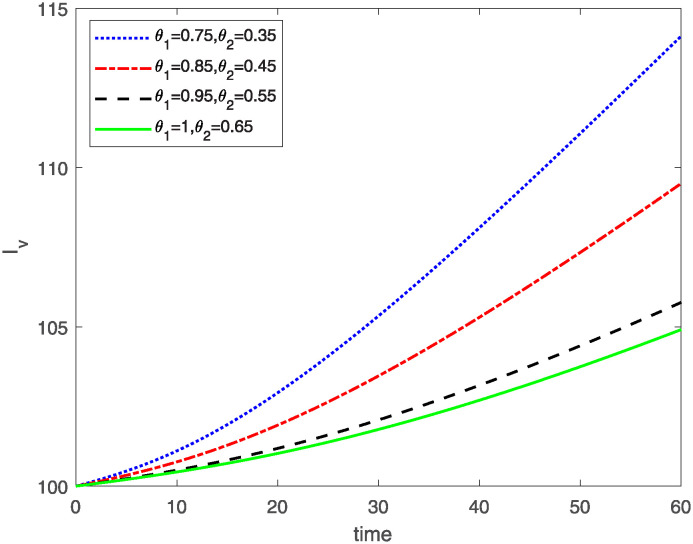
Plot for class $I_{v}\left(t\right)$ for different values of *θ*_1_*θ*_2_.

In order to generate numerical simulations, we examined a FF system (1) in this study using the Caputo derivative framework. It can be difficult to determine or choose suitable parameter values for a fractional dynamical system, as it might not be easy to measure these values precisely. Therefore, we provide the proper parameter values for the fractional PWD model (1) in this section. In our current study, we employ the following parameter values for the FF model under investigation:

This study investigates a PWD model by applying the FF Caputo derivative method to derive numerical and graphical results. We have utilized the approximate parameter values as provided in Table 1. Our simulations have encompassed various compartments of the system (1) These simulations show consistent FO *θ*_1_ and FD *θ*_2_ values while also exploring different FO and FD. Figs 2 to 13 depict the results of numerical simulations conducted using the proposed model, each corresponding to different values of parameter *θ*_1_ and *θ*_2_, specifically, 0.75, 0.85, 0.95, and 1.0. In the context of the PWD model (1), the parameters are vital for understanding the dynamics of the system, which involves interactions and processes related to pine trees, vectors, and nematodes. Figs 2–7 shows the behavior of population of $S_{h}\left(t\right)$ susceptible, $E_{h}\left(t\right)$ exposed and $I_{h}\left(t\right)$ infected pine trees population at time *t*. Similarly, Figs 8–13 shows the behavior of $S_{v}\left(t\right)$ susceptible pine trees, $E_{v}\left(t\right)$ exposed, and $I_{v}\left(t\right)$ infected pine trees population vectors at time *t*. The initial conditions and parameter values used for the simulation results are detailed in in Preliminaries section. The physical interpretation of the individual state variables of the model under the Caputo fractional operator, along with a comparison with the fractal fractional operator, is illustrated in Figs 2, 4 and 6. The fractional order values *θ*_1_ for 0.75, 0.85, 0.95, for the Caputo fractional operator, have been compared with the fractal fractional with similar values in Figs 2, 4 and 6. By analyzing the behavior of these three populations over time, one can gain insights into how the disease spreads and impacts the pine tree population. This data holds significance in comprehending the disease’s dynamics, evaluating the efficacy of control strategies, and making informed decisions for disease management and prevention. These parameters are essential for modeling the spread and dynamics of PWD within the pine tree population and the involvement of vectors and nematodes in the transmission process. By studying how these populations change over time, researchers can gain insights into disease dynamics and potential control strategies. In Figs 7–13, we show the effect of fractal parameter *θ*_1_ and fractional parameter *θ*_2_ on susceptible exposed and infected populations. For instance, increasing the fractal dimension allows us to capture the spatial heterogeneity of urban environments, while adjusting the fractional order modulates the influence of memory effects and long-term dependencies on disease spread. The present study equips public health departments with valuable tools and knowledge to mitigate the spread of Pine Wilt Disease. By leveraging advanced computational techniques and mathematical modeling, public health officials can make informed decisions and implement effective strategies to protect pine tree populations and preserve forest ecosystems.

**Table 1:** The current study utilizes these parameter values for the FF model under consideration [42].

Parameters	Values	Initial parameter	Initial values
Ξ_*h*_	0.9 [42]	$S_{h}\left(0\right)$	1000 [42]
*M*_1_	0.01 [42]	$S_{v}\left(0\right)$	500 [42]
*M*_2_	0.000127 [42]	$E_{h}\left(0\right)$	100 [42]
Ω	0.000049 [42]	$E_{v}\left(0\right)$	200 [42]
ϒ_*h*_	0.000051 [42]	$I_{h}\left(0\right)$	50 [42]
*η*_1_	0.01 [42]	$I_{v}\left(0\right)$	100 [42]
Λ_*h*_	0.0492 [42]		
Ξ_*v*_	0.2 [42]		
*π*_1_	0.000478 [42]		
ϒ_*v*_	0.000172 [42]		
Λ	0.0007 [42]		
*η*_2_	0.004 [42]		

## 7 Conclusion

A mathematical framework elucidating the propagation of PWD within a population of varying sizes has been formulated and examined. Furthermore, recognizing the significance of fractional modeling techniques, the model originally formulated for integer-order dynamics is extended to fractional order using the widely acknowledged Caputo operator. Initially, we provided a thorough theoretical analysis of the fractional PWD model, encompassing aspects such as the existence and uniqueness of solutions. Stability analysis of the Caputo model was also conducted by applying the UHS conditions. Further, the models were solved using an efficient numerical approach such as the Adams-Bashforth method, followed by detailed simulations conducted for various fractional orders and encompassed both fractal and fractional dimensions. Moreover, it creates computational techniques for fractal-fractional, allowing for comprehensive simulations to verify theoretical results for different fractional orders (*θ*_2_) and fractal dimensions (*θ*_1_). This method is a step forward in infectious disease modeling as it provides a more realistic framework for investigating intricate dynamics and evaluating intervention tactics. This work lays the groundwork for future research, where the Caputo FF operator can be applied to other epidemiological models, including those for COVID-19, HIV/AIDS, and other diseases, to explore their complex dynamics and improve prediction and control measures. Future studies could focus on expanding this model to account for additional real-world complexities, such as heterogeneous populations, varying contact rates, or environmental factors.
